# Poor accuracy of plain radiographic measurements of prosthetic migration and alignment in total ankle replacement

**DOI:** 10.1186/s13018-015-0220-x

**Published:** 2015-05-20

**Authors:** Matthias Braito, Michael Liebensteiner, Dietmar Dammerer, Martin Krismer, Martin Pfurner, Rainer Biedermann

**Affiliations:** Department of Orthopedics, Medical University of Innsbruck, Anichstrasse 35, 6020 Innsbruck, Austria; Geometry and CAD Unit, University of Innsbruck, Innrain 52, 6020 Innsbruck, Austria

**Keywords:** Total ankle replacement, Accuracy, Radiological measurement

## Abstract

**Background:**

The rotational position of the leg substantially influences measurements in ankle radiographs after total ankle replacement (TAR). The aim of our study was to further specify the influence of different projections on radiographic parameters used to assess component position after TAR.

**Methods:**

The effect of varying degrees of internal rotation, flexion, and ab-/adduction on reference lines in anteroposterior and lateral ankle radiographs was investigated in a cadaveric TAR model. Observed variations were then compared with those found in 34 consecutive patients that received a HINTEGRA total ankle prosthesis in our department.

**Results:**

A change of rotation of 20° resulted in a variation of measured reference lines of more than 1.3 cm in anteroposterior radiographs and more than 1 cm in lateral radiographs in our experimental setting. Even higher intraindividual changes of up to 1.4 cm were observed in our in vivo series.

**Conclusions:**

The findings suggest that rotational position of the leg highly influences measurements in ankle radiographs after TAR. It further raises the question, if previously described radiographic parameters do provide accurate information for the outcome after TAR in clinical routine as suggested in literature.

## Background

Total ankle replacement (TAR) has become a remarkable treatment option for end-stage osteoarthritis of the ankle joint [1–3], but the potential risks of small improvement of range of motion, persisting pain, and low functional outcome following TAR still remains high [1, 4, 5]. Therefore, hindfoot and total ankle component alignment with regard to biomechanical contact stresses and clinical outcome have been studied extensively [6–13]. Those radiographic parameters used to investigate component malalignment [6, 12–14], subsidence [15, 16], ankle alignment [3, 11, 17, 18], and range of motion [9, 10, 19] are substantially influenced by the rotational position of the leg while taking the radiographs. The aim of our study was to specify the influence of varying radiographic projections of the ankle joint on reference lines and to compare those experimentally observed variations with those observed in in-vivo specimens.

## Methods

In the first step, we conducted an experimental study using a *post-mortem* ankle specimen to investigate the influence of different radiographic projections on radiographic parameters after TAR. In a second step, observed variations were then compared with the results of radiological measurements of a retrospectively assessed cohort of 34 consecutive patients that received a HINTEGRA total ankle prosthesis (Newdeal, Lyon, France; Integra, Plainsboro, NJ, USA) at our department between 2004 and 2007. The study has been approved by the local ethics committee, and all persons gave their informed consent prior to their inclusion in the study.

### Preparation of specimen in experimental series

**Fig. 1:**
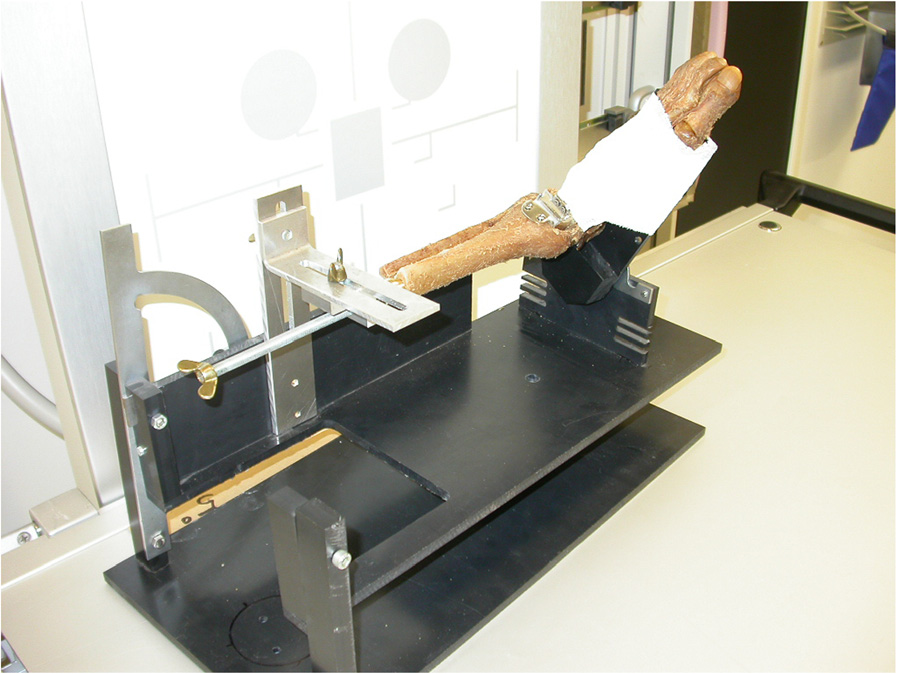
The ankle specimen fixated on the testing machine

An ankle specimen was obtained by transtibial amputation *post-mortem* from a male donor without any ankle deformity. The specimen was dissected free of soft tissue, but all major supporting ligaments were kept intact. A HINTEGRA total ankle prosthesis (tibia and talus component size 4, inlay-height 5 mm) was implanted into the ankle. The specimen was then fixated on a testing machine (Fig. 1), which allowed rotation along the longitudinal axis of the tibia and flexion/extension at a virtual axis about the knee joint. For lateral radiographs, the testing machine was positioned in a neutral or a slight ab-/adduction position of 5° to simulate for varus/valgus malpositioning of the implant.

### Measurements in experimental series

**Fig. 2:**
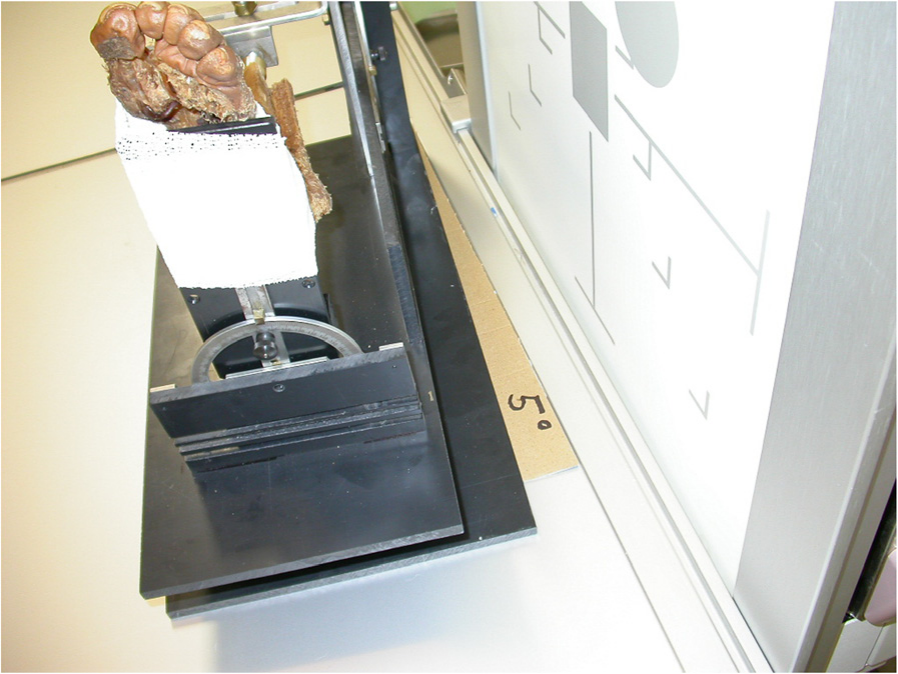
The testing machine positioned in 5° of adduction

All x-rays were obtained as digital images (Optimus SCP 80, Philipps, Amsterdam, Netherlands). We assumed a rotational variation of ±10° in real patient’s series starting from a neutral position of 10° internal rotation, which corresponds to the routine position of an ankle joint when taking radiographs at our radiological department. A series of 11 anteroposterior x-rays was obtained from 0° to 20° of internal rotation at 0° of flexion, while the ab-/adduction position was fixed. The same series of x-rays was repeated with 5° of flexion at a virtual axis about the knee joint to simulate a flexion contracture of the knee joint. Subsequently, three series of lateral x-rays were taken with the shank in 5° abduction, 0° ab-/adduction, and 5° adduction (Fig. 2) and the same range of rotation as described above to simulate for valgus/varus positioning. Consequently, we obtained 22 anteroposterior and 33 lateral x-rays in total.

**Fig. 3:**
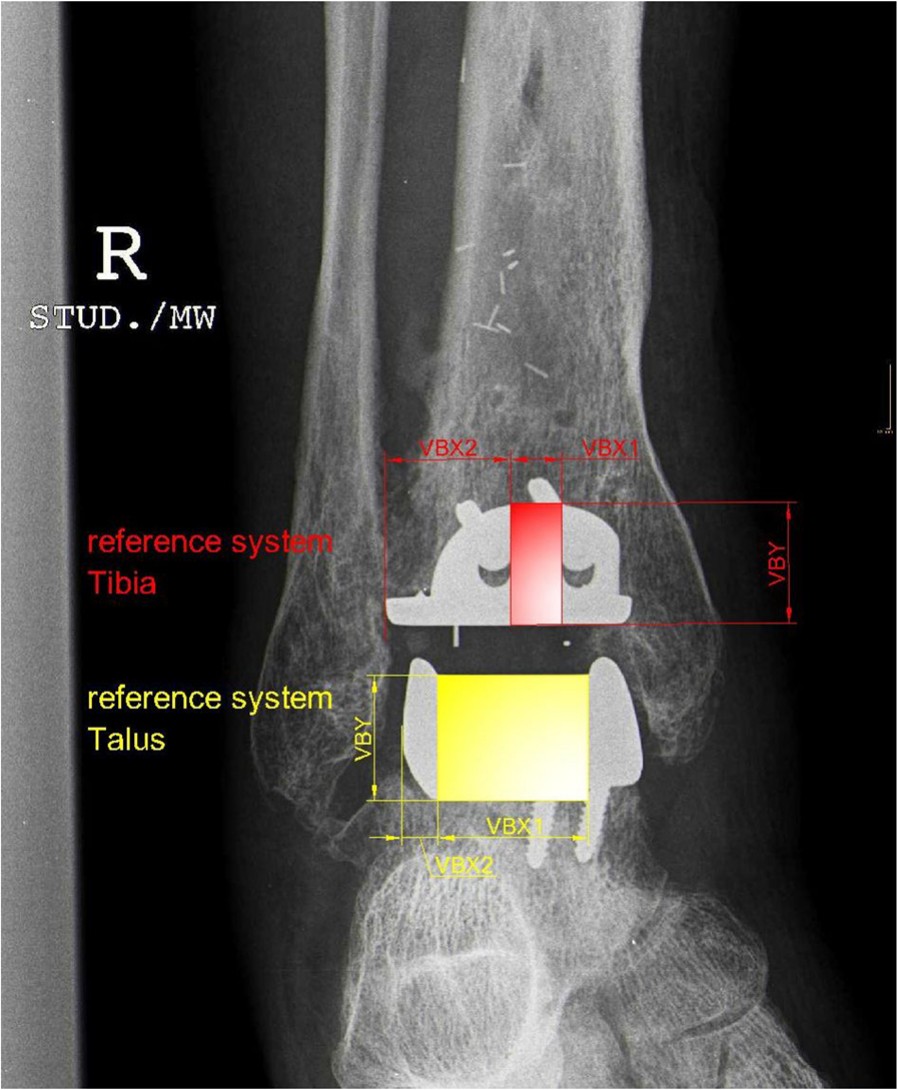
Anteroposterior reference lines

**Fig. 4:**
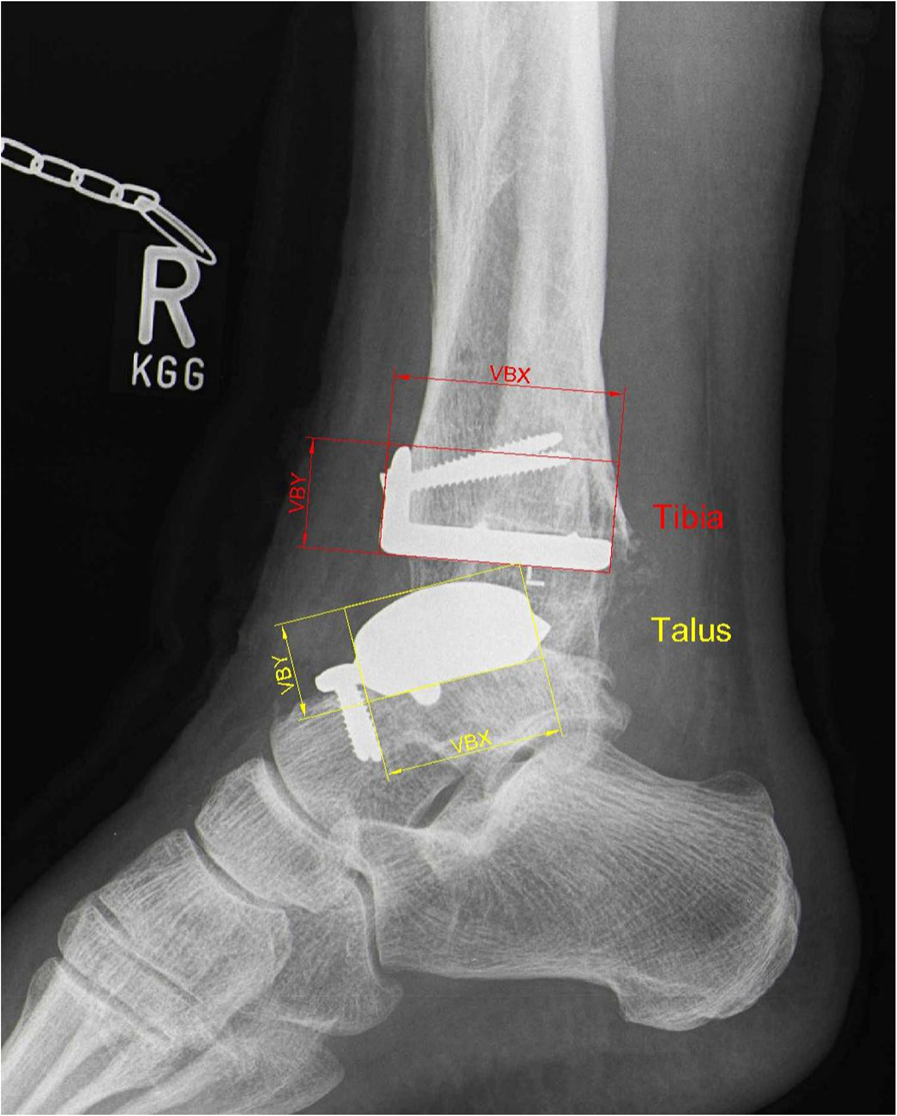
Lateral reference lines

Anteroposterior and lateral reference lines were defined to measure position and migration of the tibial and talar components (Figs. 3 and 4 show examples of specimen in plantar flexion): on the anteroposterior view, tibia_vbx1 was defined as the mediolateral distance between the medial border of the tibial component and the lateral border of the medial hole at the shield of the tibial component, tibia_vbx2 as the distance between the lateral border of the tibial component and the medial border of the lateral hole at the shield of the tibial component, tibia_vby as the maximum height of the tibial shield, talus_vbx1 as the length of the talar articulating surface, talus_vbx2 as the distance between the lateral border of the talar component and the lateral border of the talar articulating surface, and talus_vby as the height of the talar component at the articulating surface. On the lateral view, tibia_vbx was defined as the anteroposterior length of the articulating surface of the tibial component, tibia_vby as the height of the tibial shield, talus_vbx as the anteroposterior length of the articulating surface of the talar component, and talus_vby as the height of the talar component (without pegs and screws).

In a pilot study, all measurements were performed by three experienced investigators (M.L., P.M., A.E.) with the use of AutoCAD® (Autodesk, Inc., San Francisco, CA, USA) to show the intra- and interobserver reliability. All results were corrected with a magnification factor to correct for specimen-film distance (anteroposterior: magnification factor 1.29, focus-film distance 110 cm, specimen-film distance 25 cm; lateral: magnification factor 1.15, focus-film distance 110 cm, specimen-film distance 12 cm).

### In-vivo specimens

In a second step, reference lines were measured in all available standing ankle radiographs (two to eight per patient) taken in clinical routine of 34 consecutive patients that received a HINTEGRA TAR at our department between 2004 and 2007. Results were corrected with an individual magnification factor for focus-film distance (anteroposterior: magnification factor 0.9, focus-film distance 70 cm, lateral: magnification factor 0.93, focus-film distance 50 cm) and for every size of implant. Data was then compared with the experimental data.

## Results

### Experimental series

**Fig. 5:**
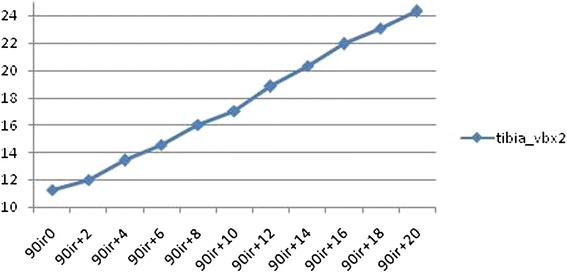
Change of tibia_vbx2 at 90° flexion in anteroposterior radiographs

**Fig. 6:**
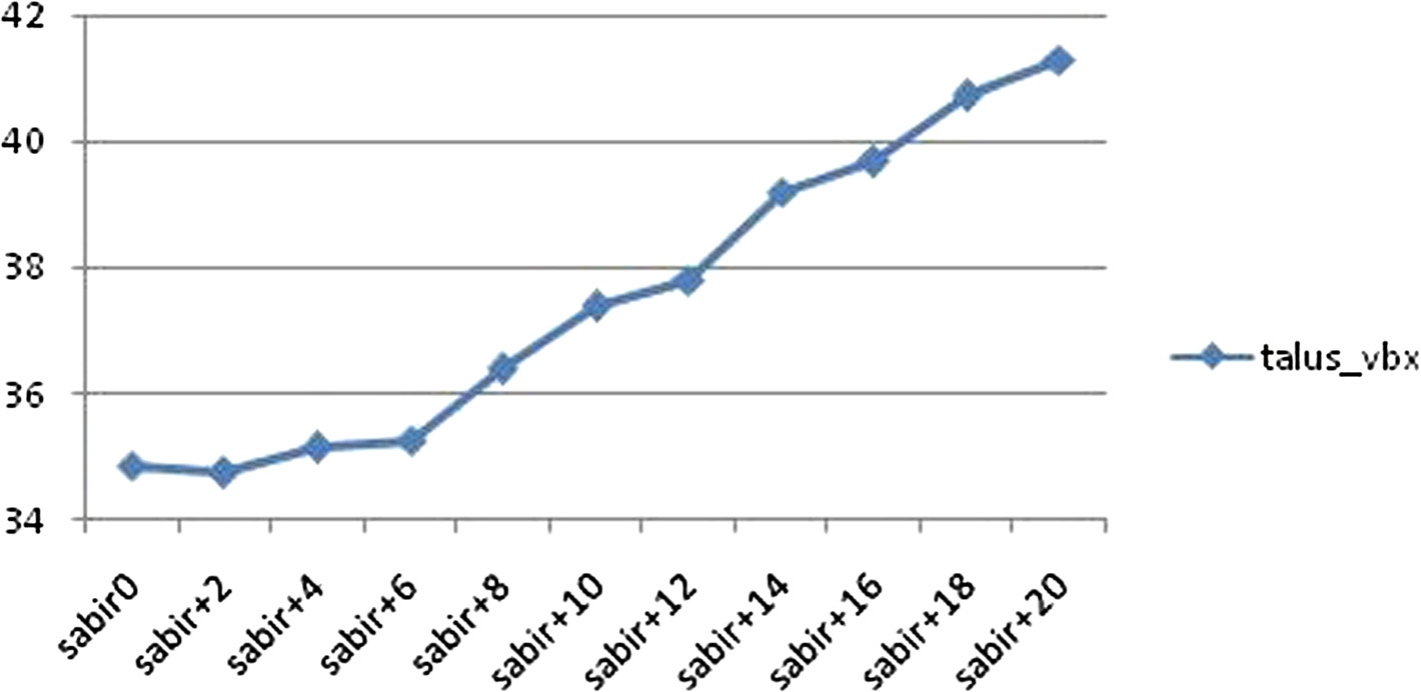
Change of talus_vbx at 5° abduction in lateral radiographs

Results of measured reference lines measurements in anteroposterior and lateral radiographs are shown in Tables 1 and 2. In the experimental setting, the maximum observed difference was 13.67 mm (tibia_vbx2) in anteroposterior x-rays and 10.75 mm (talus_vbx) in lateral x-rays. A change of rotation from 0° to 20° of internal rotation resulted in a maximum change of 13.02 mm (tibia_vbx2 at 90° flexion; Fig. 5) in anteroposterior radiographs and in a maximum change of 6.56 mm (talus_vbx at 5° abduction; Fig. 6) in lateral radiographs. Flexion of 5° about a virtual knee axis resulted in a maximum range of 1.37 mm (talus_vbx2) in anteroposterior radiographs. A change of ab-/adduction of 10° (from 5° of abduction to 5° of adduction) resulted in a maximum difference of 10.37 mm (talus_vbx) in lateral radiographs.

**Table 1:** Observed differences in anteroposterior measurements of experimental series

		tibia_vbx1	tibia_vbx2	tibia_vby	talus_vbx1	talus_vbx2	talus_vby
0°–20° internal rotation, 85° flexion	Max	11.42	23.32	20.25	27.41	8.39	16.44
	Min	10.79	10.64	18.85	21.60	3.41	16.13
	Max-min	0.63	12.68	1.41	5.81	4.97	0.32
	Mean	11.11	16.54	19.72	25.18	5.23	16.22
0°–20° internal rotation, 90° flexion	Max	11.62	24.31	25.11	28.18	9.87	18.74
	Min	10.92	11.29	23.71	20.84	4.04	18.04
	Max-min	0.70	13.02	1.40	7.34	5.83	0.70
	Mean	11.35	17.53	24.54	25.51	6.11	18.39
85°–90° flexion	Max	0.62	1.48	5.31	1.07	1.48	2.60
	Min	0.05	0.65	4.59	0.11	0.11	1.60
	Max-min	0.57	0.83	0.72	0.97	1.37	1.01
	Mean	0.30	0.99	4.82	0.52	0.88	2.17
0°–20° internal rotation, 85°–90° flexion	Max	11.62	24.31	25.11	28.18	9.87	18.74
	Min	10.79	10.64	18.85	20.84	3.41	16.13
	Max-min	0.82	13.67	6.26	7.34	6.46	2.61
	Mean	11.23	17.04	22.13	25.35	5.67	17.31

**Table 2:** Observed differences in lateral measurements of experimental series

		tibia_vbx	tibia_vby	talus_vbx	talus_vby
0°–20° internal rotation, 0° ab-/adduction	Max	48.22	20.68	39.46	22.64
	Min	45.99	17.70	34.26	17.60
	Max-min	2.22	2.98	5.21	5.04
	Mean	46.62	19.18	36.30	20.13
0°–20° internal rotation, 5° abduction	Max	48.82	19.31	41.30	20.55
	Min	46.42	18.38	34.73	17.83
	Max-min	2.40	0.93	6.56	2.73
	Mean	47.11	18.79	37.49	18.67
0°–20° internal rotation, 5° adduction	Max	49.47	22.84	34.31	26.01
	Min	47.26	20.18	30.55	21.16
	Max-min	2.20	2.66	3.77	4.85
	Mean	47.96	21.44	31.75	23.71
5° abduction-5° adduction	Max	1.59	3.94	10.75	6.13
	Min	0.02	0.20	0.02	0.24
	Max-min	1.57	3.73	10.73	5.88
	Mean	0.89	1.96	3.83	3.37
0°–20° internal rotation, 5° abduction-5° adduction	Max	49.47	22.84	41.30	26.01
	Min	45.99	17.70	30.55	17.60
	Max-min	3.47	5.14	10.75	8.40
	Mean	47.54	20.12	34.62	21.19

### In vivo specimens

Observed differences of measured reference lines in anteroposterior and lateral radiographs are shown in Table 3. The maximum change observed was 13.71 mm (tibia_vbx2) in anteroposterior x-rays and 14.27 mm (talus_vbx) in lateral radiographs.

**Table 3:** Observed differences in anteroposterior and lateral measurements of in vivo specimens

		tibia-vbx1	tibia-vbx2	tibia-vby	talus-vbx1	talus-vbx2	talus-vby
Antero-posterior	Max	2.52	13.71	7.13	11.91	9.23	6.69
	Mean	0.81	5.12	2.64	3.75	2.99	2.35
		tibia-vbx	tibia-vby	talus-vbx	talus-vby		
Lateral	Max	10.79	5.42	14.27	7.63		
	Mean	4.53	1.90	4.92	2.08		

## Discussion

In summary, our study clearly showed that reference lines used for measurement of migration and orientation of components after TAR highly depend on the rotational position of the foot while taking ankle radiographs. A change of internal rotation of 20° in our experimental series resulted in a maximum change of 1.3 cm in anteroposterior radiographs and 0.7 cm in lateral radiographs of the chosen reference lines. We assume difficulties in reproducibly positioning the ankle for a standing radiograph in clinical practice as we found even higher differences of the chosen reference lines in our real patients’ series. Therefore, measured variations might be even larger than the value to be determined itself. Assessment of components’ migration after TAR might be particularly critical, because a change of prosthesis’ position due to different projections may be misinterpreted as a sign of loosening. This might unnecessarily necessitate further investigations such as computed tomography and single photon emission computed tomography scans [20–22] or even revision surgery.

The effect of different rotational projections on consecutive radiological measurements has already been investigated in other orthopedic fields [23–29]. Significant changes in the width of the proximal femoral canal with femoral rotation have been reported [23–25]. A shift of the weight-bearing line was described with varying rotation of the foot while taking whole leg radiographs [27] and between supine and standing full-length radiographs [28]. Moreover, acromial morphology was also found to be dependent on the radiographic projection [29]. In contrast, the effect or different rotational projections in consecutive radiographs after arthroplasty has not specifically addressed so far.

Radiographic measurements further depend on accurate identification of reference points. For our study, we chose simply identifiable reference points on a total ankle prosthesis. The variability of measurements might be even higher using bony landmarks, as they might be blurred and not clearly recognizable with changing rotation or due to periprosthetic ossifications and osteoarthritic changes of adjacent hindfoot joints [30].

On the other hand, most studies dealing with radiographic measurements consider even very small differences in length or degrees of angle as significant [3, 6, 12, 31–34]. This raises the question, if radiographic parameters as suggested in literature do provide reliable information on outcome after TAR in clinical routine.

Pyevich et al. [31] evaluated the radiological and clinical outcome of 98 Agility TAR and defined three angular and one linear value to identify migration of the components. Significantly higher rates of pain were found with tibial components placed in more than 4° of valgus and in 19 cases of migration of prosthetic components. Migration was defined as a change in radiographic position of a component of more than 5°. Reliability of the measurements was tested by “having the same observer […] repeat the measurements on 60 sets of radiographs with no markings on two separate occasions” and high level of intra-rater reliability was found. However, rotation of the limb was not controlled between serial examinations and accuracy was not tested.

Barg et al. [6] reported a significant superior clinical outcome with respect to pain, AOFAS hindfoot score and ankle ROM in 127 (34.5 %) of 317 osteoarthritic ankles after HINTEGRA TAR with the center of the talar component positioned precisely in the line of the longitudinal axis of the tibia (anteroposterior offset ratio = 0) in lateral ankle radiographs taken with the patient in a standing weight-bearing position. To ensure true lateral radiographs, fluoroscopy was used for all postoperative radiographic examinations. Interobserver and intraobserver reliability was said to be good to excellent for their measurement method. However, no information about accuracy of those measurements is provided.

Hintermann et al. [32] observed migration of two talar components of 122 total ankle replacements measuring the perpendicular distance from the talar component to a line drawn between the dorsal aspect of the talonavicular joint and the calcaneal tubercle. They used fluoroscopy to ensure standardized and true anteroposterior and lateral views of both components. Again, accuracy of these measurements in standard ankle radiographs has not been shown.

Tochigi et al. [33, 34] identified a tibial-axis-to-talus ratio (T-T ratio) as a reliable and valid radiographic measure of anteroposterior tibial-talar alignment that tolerates perturbations of ankle positioning comparably best. They took lateral radiographs of ten cadaver ankles in varying ankle positions in nine prespecified positions in the transverse plane (−20° to 20° of internal rotation) and in seven positions in the sagittal plane (−10° to 20° of plantar flexion). The low 10° rotational error in the transversal plane of 2.1 % was explained by the centricity of the chosen anatomical landmarks.

On the basis of these findings, Lee et al. [12] studied the effect of anterior translation of the talus relative to the tibia using the tibiotalar ratio (TTR) and found no difference in clinical outcome after TAR between a group with a TTR within two standard deviations and group with excessive preoperative anterior talar translation (TTR of less than 29 %). They did not provide information on their used radiographic protocol.

Wood et al. [3] demonstrated that 40 (87 %) of 46 ankles with an anterior translated talus showed an improvement of the TTR after STAR total ankle replacement. The TTR correlated significantly with the inclination of the tibial component. The authors ensured that the ankle was in a similar position on the two radiographs being compared, but no information about accuracy and reliability was provided.

Our data is concordant with those of Tochigi et al. [33, 34] and clearly shows that radiographic measurements of prosthetic migration and alignment highly depend on reproducible imaging technique with correct positioning of the foot. However, in their study, Tochigi et al. might have underestimated the variability of the positioning of the foot when taking radiographs in clinical routine as they did not simulated for varying positions of ab-/adduction.

One limitation of the present study is that we were not able to simulate a real ab-/adduction or flexion/extension of the ankle joint itself due to the rigidity of the obtained ankle specimen. We do not think that this has a big impact on our main message which is the variability of radiographic reference lines. Furthermore, we did not examine the effect of rotation on commonly used markers for alignment of TAR. However, those alignment measurements are underlying a rotational variability to the same extent as the chosen reference lines in our study. Moreover, we did not use a reference ball for calibration, and prosthetic migration/subsidence in our real patient’s series might have affected our measurements. Given the short time period between those radiographs, we do not think that this influences our results to a great extent.

## Conclusions

In conclusion, accuracy of most radiographic parameters after TAR suggested in literature has not been tested yet. Our results indicate that measurement errors are likely to be expected. This might lead to wrong interpretation despite a high intra- and interobserver reliability and the use of fluoroscopy. Further studies will have to test for accuracy of suggested radiological parameters, before conclusions about outcome after TAR can be drawn.

## Abbreviations

AOFAS: American Orthopaedic Foot and Ankle Society
Fig.: figure
ROM: range of motion
TAR: total ankle replacement
tibia_vbx1: mediolateral distance between the medial border of the tibial component and the lateral border of the medial hole at the shield of the tibial component (anteroposterior view)
tibia_vbx2: distance between the lateral border of the tibial component and the medial border of the lateral hole at the shield of the tibial component (anteroposterior view)
tibia_vby: maximum height of the tibial shield (anteroposterior view)
talus_vbx1: length of the talar articulating surface (anteroposterior view)
talus_vbx2: distance between the lateral border of the talar component and the lateral border of the talar articulating surface (anteroposterior view)
talus_vby: height of the talar component at the articulating surface (anteroposterior view)
tibia_vbx: anteroposterior length of the articulating surface of the tibial component (lateral view)
tibia_vby: height of the tibial shield (lateral view)
talus_vbx: anteroposterior length of the articulating surface of the talar component (lateral view)
talus_vby: height of the talar component (without pegs and screws lateral view)
